# Awareness Level, Knowledge and Attitude Among Lebanese Medical Students Towards Interprofessional Collaboration Between Healthcare Professionals: A Cross-Sectional National Study

**DOI:** 10.7759/cureus.99445

**Published:** 2025-12-17

**Authors:** Solay Farhat, Zeinab Hammoud, Ralph Maatouk, Muhammad Barakat, Jana Kotaich, Ahmad Abou Chakra, Joe Chidiac, Anthony Mechleb, Pascale Salameh

**Affiliations:** 1 Faculty of Medical Sciences, Lebanese University, Beirut, LBN; 2 Faculty of Medicine, Caucasus International University, Tbilisi, GEO; 3 Department of Research, MEDICA Research Investigation, Hadath, LBN; 4 Department of Primary Care and Population Health, University of Nicosia Medical School, Nicosia, CYP; 5 Department of Public Health, Institut National de Santé Publique, Epidémiologie Clinique et Toxicologie (INSPECT-LB), Beirut, LBN; 6 Gilbert and Rose-Marie Chagoury School of Medicine, Lebanese American University, Beirut, LBN

**Keywords:** healthcare roles awareness, interprofessional collaboration, interprofessional education, interprofessional teams, medical education

## Abstract

Interprofessional collaboration (IPC) is essential for effective, patient-centered healthcare, requiring coordinated efforts among diverse health professionals. Interprofessional education (IPE) prepares medical students to understand their own roles and those of their colleagues, fostering communication, teamwork, and mutual respect. In Lebanon, IPE integration remains limited, and students’ awareness, knowledge, and attitudes toward IPC are underexplored. This cross-sectional national study was conducted from December 2023 to December 2024 among 608 medical students from all accredited Lebanese medical schools. Participants completed an online questionnaire assessing awareness of healthcare professionals’ roles, familiarity with IPC/IPE, and self-perceived competencies using the IPEC (Interprofessional Education Collaborative) Competency Self-Assessment Tool and the PACT (Performance Assessment Communication and Teamwork) toolkit. The mean awareness score of healthcare professionals’ roles was 6.05±1.83/10, with the highest recognition for physicians (91.2%) and psychologists (78.8%) and the lowest for physiotherapists (15.7%) and pharmacists (42.2%). Approximately 65.3% of students were willing to engage in IPE, preferring simulated patient care scenarios and case-based discussions. Clinical exposure, academic year, and hospital type significantly influenced awareness, while multivariable analyses identified willingness to participate in IPE and clinical rotation experience as the strongest predictors. Higher self-assessed IPEC competency scores were associated with increased IPC engagement. These findings highlight that Lebanese medical students demonstrate moderate awareness of IPC principles, with notable gaps in understanding non-physician roles and communication skills. Clinical exposure and positive attitudes toward IPE enhance awareness, yet competency-based training is critical to promote meaningful engagement. Integrating structured, longitudinal IPE into medical curricula is recommended to develop a collaborative, team-oriented healthcare workforce capable of delivering high-quality, patient-centered care.

## Introduction

In the continuously evolving landscape of healthcare delivery, interprofessional collaboration (IPC) has become a fundamental component of effective, patient-centered care [1]. With the growing complexity of medical conditions, technological advancements, and the increasing need for holistic treatment plans, it is no longer feasible for a single healthcare professional to manage a patient's care in isolation. Instead, optimal health outcomes are achieved through coordinated efforts of multidisciplinary teams, where professionals from diverse backgrounds - medicine, nursing, pharmacy, physiotherapy, nutrition, psychology, and radiology - work synergistically by pooling their expertise, communicating effectively, and respecting each other’s roles [2].

Interprofessional collaboration has been associated with numerous benefits, including reduced medical errors, improved patient satisfaction, enhanced health outcomes, more efficient use of healthcare resources, and increased job satisfaction among healthcare providers [3,4]. These advantages are supported by a growing body of evidence from international settings where team-based care models have been successfully integrated into clinical practice. For instance, the World Health Organization (WHO) has long advocated for interprofessional education (IPE) as a strategy to prepare a collaborative, practice-ready health workforce that can meet complex patient needs in a rapidly changing world [5].

IPE emerges as a key catalyst in preparing healthcare professionals for collaboration in their future endeavors [6]. By providing a platform for individuals to understand and embrace the intricacies of working with professionals from various fields, IPE lays the foundation for a collaborative healthcare environment. However, despite its transformative potential, there remains a notable gap in healthcare personnel’s communication strategies and their understanding of their own roles and those of their colleagues [7]. This deficiency is often attributed to the inadequacy of undergraduate education on collaborative practices and the insufficient training of physicians in this regard.

Traditional models of medical education tend to be profession-centric, often conducted in silos that limit interaction with students from other healthcare disciplines [8]. This isolation hinders the development of essential competencies such as teamwork, communication, and mutual respect - skills foundational to successful IPC. Without adequate exposure to IPE during their formative academic years, medical students may enter the clinical workforce with a limited understanding of the contributions and responsibilities of their peers from other health professions. This not only affects team cohesion but can also lead to fragmented care, inefficiencies, and professional tension.

The existing gap in knowledge particularly affects medical students' awareness of the roles and responsibilities of other healthcare professionals. The failure to instill a strong foundation in collaboration during the early stages of training can hinder effective communication and teamwork within healthcare teams. Bridging this knowledge gap is imperative to fostering a healthcare workforce that seamlessly integrates the expertise of diverse professionals, ultimately enhancing patient care and healthcare outcomes [9].

In the Lebanese context, the integration of IPE remains in its infancy. Most medical curricula in Lebanon continue to follow traditional pedagogical models, where students are primarily exposed to discipline-specific training. While clinical rotations may offer opportunities for interaction with various healthcare professionals, these encounters are often unstructured and incidental rather than intentionally designed to foster interprofessional learning. Consequently, many Lebanese medical students may graduate with minimal awareness of IPC principles and the collaborative competencies required in modern healthcare settings. Given the increasing demands placed on the Lebanese healthcare system - exacerbated by recent socioeconomic and infrastructural challenges - strengthening teamwork and collaboration across disciplines is more urgent than ever.

Understanding how medical students perceive and engage with IPC is critical for informing curriculum reform and policy development. Medical students, as future physicians and potential leaders in healthcare, play a pivotal role in shaping the culture and practices of the clinical environments in which they will work. Fostering early awareness, knowledge, and positive attitudes towards IPC is essential to cultivating a collaborative ethos that can ultimately enhance patient care outcomes across the country.

With that being stated, this study primarily aims to assess the awareness, knowledge, and attitudes of Lebanese medical students toward IPC. While focusing on their understanding of the roles and responsibilities of non-physician healthcare professionals, including nurses, imaging technicians, pharmacists, dietitians, physiotherapists, psychologists, and physicians from other specialties, this study also examines how factors such as gender, academic year, and clinical exposure influence these perceptions. By identifying gaps in knowledge and attitudes, the research seeks to support the development of targeted educational interventions that strengthen interprofessional competencies. Ultimately, it contributes to the ongoing discourse on healthcare education reform in Lebanon, advocating for the integration of structured interprofessional education to foster a collaborative, team-oriented healthcare workforce.

## Materials and methods

Study design and population

This cross-sectional prospective study was conducted among medical students in Lebanon over a one-year period from December 2023 to December 2024. Participants were recruited using a snowball sampling technique [10] via an online, self-administered English-language questionnaire distributed through university networks and social media platforms. Only students who completed the full questionnaire and consented to participate were included in the analysis.

Participants

Eligible participants were medical students currently enrolled in medical schools across Lebanon, aged 18 years or older, and residing in Lebanon at the time of the study. Exclusion criteria included physicians, nurses, and other healthcare professionals already practicing in the field, as well as other and non-healthcare students (e.g. nursing students) or medical students living outside Lebanon. Participants who did not provide informed consent were also excluded.

Sample size determination

Using Slovin’s formula [11], $n = \frac{N}{1 + N e^2}$, with a total population of approximately 3,900 medical students in Lebanon and a margin of error of 5%, the minimum required sample size was calculated to be approximately 363 participants. In addition, the minimum sample size needed for the multivariable analysis was calculated using G*Power software version 3.1.9.7 for Windows (developed by Franz Faul, Edgar Erdfelder, Albert-Georg Lang, and Axel Buchner; Heinrich Heine University, Düsseldorf, Germany).

Assuming a small effect size of $f^2 = 0.05$ and anticipating a squared multiple correlation of 0.05 ($R^2$ deviation from 0) for the omnibus test of multiple regression, the required sample size was determined to be n=415, based on an alpha error of 5%, a power of 80%, and the inclusion of up to 20 predictors in the model. The actual sample size obtained in this study (n=608) exceeded both requirements.

Data collection tool and variables

The questionnaire collected demographic data including age, gender, university, and year of study. It also included four main components assessing students’ awareness and attitudes toward interprofessional collaboration and the roles of other healthcare professionals:

Awareness of Roles and Responsibilities of Other Healthcare Professionals

This section evaluated knowledge of the roles of various health professionals including nurses, pharmacists, physiotherapists, psychologists, dietitians, imaging technicians, and specialists. Questions were developed using practical examples from hospital settings and supported by existing literature [12,13].

Knowledge and Attitudes Toward Interprofessional Collaboration (IPC) and Interprofessional Education (IPE)

Questions in this section assessed participants’ familiarity with and openness to IPC and IPE concepts, aiming to evaluate their preparedness for collaborative practice. A validated scale was used to measure self-assessed competencies related to communication, teamwork, professional roles, and values/ethics within interprofessional healthcare settings: the IPEC (Interprofessional Education Collaborative) Competency Self-Assessment Tool - Version 3 [14].

Performance Assessment - Communication and Teamwork

This section, based on the PACT (Performance Assessment Communication and Teamwork) toolkit (Center for Health Sciences, Interprofessional Education, Research, and Practice, University of Washington), evaluated students’ perceived abilities in interprofessional communication and team collaboration [15].

Ethical considerations

The study was conducted in accordance with the principles outlined in the Declaration of Helsinki. Ethical approval was obtained from the appropriate institutional review board (IRB). Prior to participation, respondents were informed of the study’s purpose, procedures, and their rights. Consent was obtained electronically. Participation was voluntary and anonymous. No incentives were offered, and data was kept strictly confidential for research purposes only.

Data analysis

Data was analyzed using IBM SPSS Statistics, version 28.0 (IBM Corp, Armonk, NY). Descriptive statistics (frequencies, percentages, means, standard deviations) were used for demographic and categorical variables. Median and interquartile ranges were presented for non-normally distributed continuous variables. The normality of distributions was evaluated through histograms, skewness, and kurtosis.

Validity and reliability of the scales were assessed using exploratory factor analysis, Spearman’s coefficients, and Cronbach’s alpha. For bivariate analysis, Student’s t-test and analysis of variance (ANOVA) were used for normally distributed variables, with Levene’s test for homogeneity of variances. Non-parametric tests (Kruskal-Wallis and corrected t-tests) were used as appropriate, with Bonferroni adjustment for post-hoc comparisons. Chi-square and Fisher’s exact tests were used for categorical data.

Multivariable analysis included multiple linear regression for continuous outcomes and logistic regression for dichotomous outcomes, with model assumptions verified (normality, linearity, multicollinearity, and homoscedasticity). Variables with p-values <0.1 in bivariate analysis and those considered of practical value were included in the models, ensuring variable-to-sample size ratio was maintained. Model adequacy was confirmed using appropriate assumptions. The inclusion criteria for predictors ensured an appropriate variable-to-sample size ratio and improved reproducibility of the analysis.

## Results

Demographic characteristics of the participants

Out of a total of 608 medical students from all accredited medical schools in Lebanon, the mean age was 21.76±1.69 years (participating range: 18-30 years). The gender distribution included 359 (59.1%) women and 249 (40.9%) men. The majority of respondents were single (n=599, 98.5%) and only nine (1.5%) participants were married. Regarding the type of institution, 312 (51.3%) students were from the Lebanese University, the only public university in Lebanon, while 296 (48.7%) were from private universities, including the American University of Beirut (AUB), the Lebanese American University (LAU), the University of Balamand (UoB), Beirut Arab University (BAU), Saint Joseph University (USJ), Holy Spirit University of Kaslik (USEK), and Saint George University of Beirut (SGUOB).

In terms of academic exposure, 122 (20.1%) students were currently in their clinical years, 201 (33%) had completed at least one clinical rotation despite being in preclinical years, and 285 (46.9%) had no clinical exposure at all. Out of the participants with clinical experience, the majority were trained in private hospitals (n=189, 58.6%), while 33 participants (10.2%) had their training in a governmental hospital. Notably, 77 participants (23.8%) had received training from both private and governmental hospitals.

Only 82 (13.5%) students reported completing clinical rotations abroad, while 526 (86.5%) had no international exposure. This distinction was reported to explore whether exposure to clinical training abroad may be associated with a more developed understanding of interprofessional collaboration compared to training received locally. Table 1 summarizes participant demographics and clinical exposure characteristics.

**Table 1:** Demographic Characteristics and Clinical Exposure of Participants (N=608)

Variable	N (%)
Gender	
Female	359 (59.1%)
Male	249 (40.9%)
Marital status	
Single	599 (98.5%)
Married	9 (1.5%)
Type of university of medical education	
Public university	312 (51.3%)
Private university	296 (48.7%)
Clinical rotation involved	
Completed rotation	323 (53.1%)
Did not complete rotation	285 (46.9%)
Type of hospital trained in	
Trained in private hospital	189 (58.6%)
Trained in public hospital	33 (10.2%)
Trained in both	77 (23.8%)
Not specified	24 (7.4%)
Rotation abroad	
Completed rotation abroad	82 (13.5%)

Knowledge of healthcare professionals’ roles

Participants answered a 10-item questionnaire assessing their understanding of healthcare professionals' roles. The mean awareness score was 6.05±1.83 out of 10. The highest correct recognition was for roles of physicians (91.2%) and psychologists (78.8%), while physiotherapists (15.7%) and pharmacists (42.2%) roles had the lowest recognition. Table 2 presents role-specific scores.

**Table 2:** Awareness of Healthcare Professionals’ Roles (N=608)

Profession	% Correct	% Incorrect
Physician	91.2	8.8
Psychologist	78.8	21.2
Dietitian	54.4	45.6
Nurse	53.1	46.9
Radiological technician	49.3	50.7
Pharmacist	42.2	57.8
Physiotherapist	15.7	84.3

Attitudes and willingness toward interprofessional education (IPE)

Out of 608 students, 397 (65.3%) expressed willingness to participate in IPE, while 211 (34.7%) were unwilling. Regarding preferred activities, simulated patient care scenarios were selected by 333 students, and case-based discussions by 317 students.

Self-Assessed Interprofessional Competency - IPEC Competency Self-Assessment Tool

The IPEC Self-Assessment Tool was used to measure Lebanese medical students' self-perceived interprofessional competencies across two domains: Interprofessional Interactions (items 1-8) and Interprofessional Values (items 9-16). Overall, students rated themselves between 3.7 and 4.1 on a five-point Likert scale, reflecting a perception of being “competent” to “somewhat advanced” across most competencies. The Interprofessional Interaction domain yielded a mean score of 3.87±0.80, while the Interprofessional Values domain scored slightly higher at 4.02±0.84, suggesting that students expressed greater confidence in professional values such as integrity and respect compared to day-to-day collaborative and communication skills.

Performance Assessment: Communication and Teamwork

In reference to the PACT self-assessment, which comprises two domain-Interaction, reflecting competencies related to communication, teamwork, and collaborative behaviors with other health professionals, and Values, capturing attitudes such as respect, integrity, and ethical responsibility in interprofessional practice-students reported moderate to high levels of perceived competency.

Notably, scores for the Interprofessional Interaction domain ranged from 3.73 to 4.03, with an overall mean of 3.87±0.80. The highest-rated item was “I am able to understand the responsibilities and expertise of other health professions” (mean=4.03, SD=0.93), indicating strong awareness of the roles of other healthcare professionals, whereas the lowest-rated item was “I am able to choose communication tools and techniques that facilitate effective team interactions” (mean=3.73, SD=0.99), pointing to a need for more structured training in communication strategies.

On the other hand, the Interprofessional Values domain scored slightly higher, with means ranging from 3.88 to 4.11 and an overall mean of 4.02±0.84. The highest-rated item was “I am able to act with honesty and integrity in relationships with other team members” (mean=4.11, SD=0.92), highlighting students’ strong ethical foundation, while the lowest-rated item was “I am able to place the interests of patients and populations at the center of interprofessional health care delivery” (mean=3.88, SD≈0.90), underscoring the need for greater emphasis on patient-centered interprofessional practice.

Bivariate Analysis

Table 3 summarizes the bivariate analysis of awareness scores across sociodemographic and academic variables. No significant gender differences were observed, as men and women reported comparable mean awareness scores (6.05±1.83; t=-0.067, p=0.947). In contrast, awareness varied significantly by academic year (F=11.68, p<0.001), with scores steadily increasing from the pre-clinical to the clinical years; sixth- and seventh-year students achieved the highest mean scores (7.12±1.52 and 7.09±1.92, respectively). University affiliation also influenced awareness (analysis of variance (ANOVA) p=0.022; Kruskal-Wallis p=0.015), with students from private universities reporting the highest mean scores.

Clinical exposure showed a particularly strong association with awareness. Students who had completed at least one clinical rotation scored significantly higher (6.50±1.79) than those without rotation experience (5.60±1.76; Welch’s t≈5.94, p<0.00001). Moreover, the number of departments rotated was positively correlated with awareness (Spearman’s ρ=0.31, p<0.00001). Students exposed to at least one high-intensity department - such as the intensive care unit (ICU), coronary care unit (CCU), internal medicine, hematology, or oncology - reported higher scores compared to their peers without such exposure (6.80±1.70 vs. 5.65±1.70; t=7.77, p<0.00001). Type of hospital exposure further shaped awareness, with students training exclusively in public hospitals achieving the highest mean scores (7.3±1.6), followed by those with mixed exposure, while those training exclusively in private hospitals had the lowest scores (6.3±1.8; ANOVA/Kruskal-Wallis p<0.001).

Finally, awareness was significantly associated with willingness to participate in IPE. Among students with high awareness scores (≥6.05), 87.4% expressed willingness to engage in IPE compared to only 45.5% of those with lower scores (χ² test, p<0.001). Collectively, these findings indicate that awareness is shaped primarily by academic progression, institutional and hospital exposure, and clinical experience (Table 3).

**Table 3:** Bivariate Analysis of Awareness Scores by Student Characteristics IPE: Interprofessional education; USJ: Saint Joseph University; USEK: Holy Spirit University of Kaslik; UoB: University of Balamand.

Variable	Mean Awareness ± SD	p-value
Gender (Male)	6.05 ± 1.83	0.947
Gender (Female)	6.05 ± 1.83	0.947
Academic Year 1	5.27 ± 1.84	<0.001
Academic Year 2	5.21 ± 1.83	<0.001
Academic Year 3	5.70 ± 1.80	<0.001
Academic Year 4	5.81 ± 1.68	<0.001
Academic Year 5	6.08 ± 1.69	<0.001
Academic Year 6	7.12 ± 1.52	<0.001
Academic Year 7	7.09 ± 1.92	<0.001
Hospital Type (Public)	7.3 ± 1.6	<0.001
Hospital Type (Private)	6.3 ± 1.8	<0.001
Hospital Type (Both)	6.8 ± 1.7	<0.001
University Affiliation (USJ)	6.9 ± 1.7	0.022
University Affiliation (USEK)	6.7 ± 1.8	0.022
University Affiliation (UoB)	6.6 ± 1.7	0.022
Clinical Rotations (Yes)	6.50 ± 1.79	<0.001
Clinical Rotations (No)	5.60 ± 1.76	<0.001
Exposure to High-Intensity Dept (Yes)	6.80 ± 1.70	<0.001
Exposure to High-Intensity Dept (No)	5.65 ± 1.70	<0.001
Awareness ≥ 6.05 (Willingness to IPE)	87.4% willingness	<0.001
Awareness < 6.05 (Willingness to IPE)	45.5% willingness	<0.001

Multivariable Analysis

A multivariable linear regression analysis was conducted to identify key predictors of medical students' awareness of interprofessional roles. The model incorporated six independent variables: gender, academic year, clinical rotation experience, type of hospital, self-assessed IPEC competency scores, and willingness to participate in interprofessional education (Table 4). The results indicated that willingness to participate in IPE was the most significant predictor, with a beta coefficient of 0.60 (95% CI: 0.42 to 0.78, p<0.001), explaining 6.61% of the variance (R²=0.0661). Clinical rotation experience also demonstrated a strong positive association (β=0.59, 95% CI: 0.39 to 0.79, p<0.001), accounting for 4.7% of the variance.

Notably, academic year and hospital type had modest contributions, with β values of 0.10 (95% CI: 0.02 to 0.18, p=0.012) and 0.6 (95% CI: 0.21 to 0.99, p=0.003), respectively, explaining less than 2% each. Conversely, IPEC competency scores and gender displayed negligible effects. Overall, these findings suggest that attitude towards IPE and clinical exposure are the primary determinants of awareness levels, emphasizing the importance of fostering positive perceptions and early experiential learning.

A logistic regression analysis was performed to evaluate factors influencing students’ willingness to engage in interprofessional education. The model identified awareness score (OR=1.83, 95% CI: 1.45 to 2.30, p<0.001), clinical experience (OR=2.07, 95% CI: 1.65 to 2.59, p<0.001), academic year (OR=1.26, 95% CI: 1.05 to 1.52, p=0.012), and self-assessed IPEC competency score (OR=1.48, 95% CI: 1.18 to 1.85, p=0.001) as significant predictors. The model explained approximately 13.3% of the variance (pseudo R²=0.1332) and achieved an overall accuracy of 68.9%. Notably, higher awareness and clinical experience significantly increased the likelihood of willingness to participate in IPE, with clinical exposure nearly doubling the odds. These results underscore that enhancing students’ awareness of interprofessional roles and providing clinical opportunities emphasizing teamwork can effectively promote engagement in interprofessional education.

Regarding interprofessional collaboration frequency, a multinomial logistic regression identified university affiliation and IPEC competency as significant determinants (Table 5). The model revealed that students with higher interprofessional competency scores were more likely to report high levels of collaboration engagement (OR=3.93, 95% CI: 2.80 to 5.52, p<0.001). University differences also significantly influenced collaboration frequency, suggesting institutional culture and curricular differences shape engagement patterns. Interestingly, awareness levels and academic year did not significantly affect collaboration frequency, indicating that practical competency development, rather than mere awareness or seniority, plays a crucial role. Overall, these findings emphasize the need for targeted, competency-based interprofessional training and institutional support to foster active collaboration among medical students.

**Table 4:** Predictors of Awareness of Interprofessional Roles (Multivariable Linear Regression) IPE: Interprofessional education; IPEC: Interprofessional Education Collaborative.

Predictor	Coefficient (β)	95% CI	p-value	R² / OR	Interpretation
Willingness to participate in IPE	0.60	0.42 to 0.78	<0.001	6.61%	Strong predictor; higher willingness increases awareness
Clinical rotation experience	0.59	0.39 to 0.79	<0.001	4.7%	Clinical exposure positively influences awareness
Academic year	0.10	0.02 to 0.18	0.012	1.45%	Slight positive effect on awareness
Type of Hospital (public vs. private)	0.60	0.21 to 0.99	0.003	1.16%	Exposure impacts awareness modestly
IPEC competency score	-0.09	-0.30 to 0.12	0.402	0.33%	No meaningful predictive value
Gender	0.057	-0.19 to 0.30	0.647	0.04%	No effect

**Table 5:** Predictors of Willingness to Participate in IPE (Multivariable Logistic Regression) IPE: Interprofessional education; IPEC: Interprofessional Education Collaborative.

Predictor	Odds Ratio (OR)	95% confidence interval (CI)	p-value
Awareness score	1.83	1.45-2.30	<0.001
Clinical experience	2.07	1.65-2.59	<0.001
Academic year	1.26	1.05-1.52	0.012
IPEC competency score	1.48	1.18-1.85	0.001

## Discussion

This study is a cross-sectional national study that offers critical insight into the current awareness, knowledge, and attitudes of Lebanese medical students toward IPC and IPE. Our data, drawn from a representative cohort of students across all accredited Lebanese medical schools, highlight both encouraging trends and persistent gaps that must be addressed to foster more effective interprofessional healthcare in the future.

The moderate awareness score of 6.05 out of 10 indicates that while students possess a general understanding of certain healthcare roles, significant misconceptions or lack of recognition persist - particularly regarding physiotherapists and pharmacists. This aligns with the broader challenges identified in the literature, where health professional roles beyond physicians and psychologists are often underrecognized [16]. Such gaps in role understanding may impede collaboration in real-world clinical settings, where the full potential of team-based care depends on mutual respect and clarity of roles among all professionals.

Notably, our study observed that clinical exposure had a substantial impact on awareness levels. Students who had participated in at least one clinical rotation - especially in high-intensity departments such as ICUs or hematology - demonstrated significantly higher awareness and knowledge scores. This supports findings from Vermeulen et al. [17], who reported that direct clinical engagement through structured IPE significantly enhanced student competence, autonomy, and collaborative perception. The correlation between exposure to real-world team-based care environments and heightened interprofessional awareness underscores the educational value of experiential learning in cultivating IPC competencies.

Indeed, the willingness of 65.2% of students to engage in IPE is promising and suggests a readiness among future healthcare providers to adopt more collaborative practices. However, the remaining 34.7% who expressed reluctance cannot be overlooked. These findings reflect a common dilemma in IPE implementation: while many students recognize the potential value of interprofessional learning, a significant proportion remain disengaged or unaware of its relevance. As noted in the meta-review by Wei et al. [18], barriers to IPC and IPE often exist at the individual level (e.g., lack of role clarity or perceived irrelevance), institutional level (e.g., curriculum overload), and organizational level (e.g., absence of IPE infrastructure). Thus, merely offering IPE opportunities may be insufficient; institutions must proactively embed IPC into the medical culture through targeted strategies that emphasize relevance, practical impact, and shared responsibility.

Furthermore, our regression analyses indicate that clinical experience and higher awareness levels are not only associated with better self-assessed competency but also significantly predict students’ willingness to engage in IPE. However, while awareness and clinical exposure were predictors of willingness, engagement in actual interprofessional collaboration was most strongly predicted by IPEC self-competency scores. This finding emphasizes that technical awareness alone does not guarantee collaborative practice; rather, the internalization of interprofessional values - particularly those related to communication and teamwork - is essential. It is important to emphasize that fostering a "culture of caring" and mutual respect is at the heart of meaningful collaboration, and our findings support this notion [19]. Students who self-reported higher scores in value-driven behaviors, such as integrity and communication, were more likely to engage in collaborative practices.

University affiliation also emerged as a significant factor influencing awareness and collaborative engagement, suggesting variability in how different institutions integrate or prioritize interprofessional learning. This is consistent with the observations of Neill et al. [20], who found that centralized and well-structured IPE infrastructures led to more consistent outcomes than decentralized or grassroots efforts. The experience of institutions that adopted system-wide IPE strategies should serve as a model for Lebanese medical schools aiming to reduce variability in IPC exposure and better align student outcomes across universities.

Interestingly, gender did not significantly affect awareness, contradicting findings from some earlier studies that reported gender-based differences in collaborative attitudes. This may reflect evolving attitudes in newer generations of medical students or a more balanced representation of genders across Lebanese medical schools.

Our results also draw attention to the particular neglect of interprofessional roles such as pharmacists and physiotherapists - professions critical to holistic patient care. A recent scoping review by Bandiera et al. highlighted how pharmacist-community health worker collaborations positively impacted patient outcomes and emphasized the need to integrate such models into broader interprofessional training [21]. The consistently low awareness of non-physician roles observed in our study calls for deliberate curriculum reform, including interdisciplinary shadowing experiences and joint workshops to demystify roles and responsibilities across professions.

The need for structured IPE in the undergraduate medical curriculum is further emphasized by findings from Pitout et al., who demonstrated the utility of curriculum mapping to identify and address gaps in IPE competency delivery [16]. Their approach of mapping IPEC core competencies - values/ethics, roles/responsibilities, communication, and teamwork - provides a useful framework that could be adapted to the Lebanese context. Our results, which show lower self-rated competency in communication tools relative to integrity or respect, echo this imbalance and highlight areas for targeted intervention.

In light of these findings, we propose a multipronged solution to enhance IPC awareness and readiness in Lebanon. First, national medical education policy should mandate the inclusion of longitudinal, structured IPE modules that expose students early and regularly to team-based care settings. Second, clinical rotations should be revised to include multidisciplinary shadowing and active participation with other healthcare professionals. Third, universities should adopt competency-based assessment tools such as the IPEC framework to evaluate and iteratively improve students' preparedness for collaborative practice.

Finally, inter-university collaborations could foster a national IPE consortium, much like the statewide model described by Neill et al. to unify efforts, share resources, and ensure equity in interprofessional education across the country [20]. Such structural reforms would not only elevate the competencies of Lebanese medical students but also align national educational practices with global standards of patient-centered, team-based care [6,22].

While many students express openness and some demonstrate strong competency foundations, notable gaps remain in knowledge, communication readiness, and institutional integration. Addressing these gaps requires both educational and cultural transformation - one that prioritizes team-based care, encourages mutual understanding, and empowers future professionals to work not in silos, but in synergy.

Limitations

While this study provides valuable insights into the awareness, knowledge, and attitudes of Lebanese medical students toward interprofessional collaboration, several limitations should be considered. First, the cross-sectional design limits our ability to establish causal relationships between exposure variables and outcomes. While associations were identified, longitudinal or interventional studies would be necessary to assess the long-term impact of interprofessional education on collaborative behavior.

Second, the use of a snowball sampling technique and online distribution of the questionnaire may have introduced selection bias. Students who are more interested or aware of interprofessional collaboration may have been more likely to participate, potentially overestimating the general level of awareness and willingness within the broader student population.

Third, despite efforts to include participants from all accredited medical schools in Lebanon, the representation from each institution may have varied, and institutional curricula or hidden curricular elements influencing IPC exposure could not be fully accounted for. Furthermore, self-reported measures, including the IPEC competency tool, are subject to social desirability bias and may not accurately reflect actual competence or collaborative behavior in clinical practice.

Lastly, although the questionnaire was based on validated tools and adapted to the Lebanese context, cultural nuances and interpretation of specific healthcare roles may vary, possibly affecting participants' responses. Future studies should consider triangulating self-reported data with observational or performance-based assessments for a more comprehensive evaluation of interprofessional competencies.

## Conclusions

Through our study, we've highlighted the urgent need to advance interprofessional education (IPE) among Lebanese medical students. While many students expressed willingness to engage in IPE and demonstrated a basic understanding of collaborative care, notable gaps remain - especially in recognizing non-physician roles and developing communication skills. Indeed, clinical exposure and academic progression were associated with higher awareness and readiness for collaboration, but self-assessed competencies in teamwork and values were the strongest predictors of actual engagement.

To address these gaps, Lebanese medical schools, as well as other regional and international institutions, should consider integrating structured, longitudinal IPE into their curricula. Doing so will foster a new generation of healthcare providers equipped with both clinical expertise and the collaborative mindset essential for delivering effective, team-based care.

## Appendices

Appendix A - Study questionnaire: awareness level, knowledge, and attitude among Lebanese medical students towards interprofessional collaboration between healthcare professionals

Section I: Consent

Hello! We're medical students from the Lebanese University, Faculty of Medical Sciences, working on our graduation thesis project. The following research study is intended for Lebanese medical students with the aim of assessing the level of awareness among medical students regarding the roles and responsibilities of various healthcare professionals. The survey will ask about demographics, awareness and willingness towards interprofessional collaboration and education, and self-assessment tools. The survey will take about five minutes to fill. Students who will be enrolled in this study are medical students in Lebanon over the age of 18 and below age 65, at all medical-years levels (residents not included), from the eight medical schools in Lebanon. If you are eligible and agree to participate in this research study, the information will be kept confidential. No harm will be caused in any possible way and protection of the privacy and anonymity of the participants will be assured. We will not be asking about any of your personal information such as phone number, or email and the data will only be used for research purposes. In addition, if you start the survey and feel that you don’t want to continue, feel free to stop at any time; your information will not be used if the survey was not completed and submitted.

1. Do you consent to participate in this study? (Yes / No)

Section II: Sociodemographic and Personal Data

2. Age (in years)

3. Gender (Male / Female)

4. Marital Status (Single / Married / Divorced / Widowed)

5. Which province do you live in? (North Lebanon / Beirut / Mount Lebanon / Beqa'a / South Lebanon / Nabatiyeh / Akkar / Baalbeck-Hermel)

6. Which medical school are you currently enrolled in? (LU / AUB / BAU / UoB / USJ / LAU / USEK / Saint George University of Beirut)

7. Academic Year (First-Seventh year)

8. Have you completed any clinical rotations or internships? (Yes / No)

9. Was your clinical rotation in a public or private hospital? (Public / Private / Both / None)

10. Did you have any clinical rotation abroad? (Yes / No)

11. Which medical departments have you completed rotations in? (Select all that apply: Internal Medicine, ER, ICU/CCU, Surgery, Pediatrics, Anesthesia, Hematology/Oncology, OB/GYN, None)

Section III: Awareness of Healthcare Professions’ Roles

12. What role does a hospital pharmacist typically play in ensuring medication safety?

·         Prescribe patient's medication

·         Adjustment of patient's medication doses

·         Checking for medication allergies and interactions

·         Performs primary management upon patient's intoxication

13. Among the following, which is a primary role of registered nurses to ensure patient advocacy and ethical care?

·         Drawing blood from a patient's vein(s) (phlebotomy)

·         Charting and documentation

·         Patient's hygiene care

·         Intubating a patient

14. True or False: A psychologist offers support in end-of-life decisions.

·         True

·         False

15. Do medical physicians working at the hospital collaborate with psychologists, psychiatrists, or both?

·         Psychologists

·         Psychiatrists

·         Both

16. Is physiotherapy present in your medical department? (Yes / No / Don’t know / None)

·         I did not complete any clinical rotation

·         Yes

·         No

·         I don't know

17. Among the following responsibilities, what might a physiotherapist collaborate on?

·         Rehabilitation of a patient pre-surgery

·         Prescribe medication

·         Provide mental assessment as well analyse strength, range of motion and mobility

·         Create personalized treatment plans, setting specific goals, and utilizing various interventions.

18. What is a critical aspect of a dietitian’s role when working with critically ill patients?

·         Monitoring nutritional status

·         Create generalized meal planning

·         Decision regarding whether a patient should receive nutrition orally (PO) or via a nasogastric (NG) tube

·         Providing nutritional support and optimizing nutrient intake

19. What is a critical responsibility of a radiologic technician during CT scans?

·         Administers contrast agents intravenously

·         Interprets the CT images for diagnostic purposes

·         Does NOT offer proper patient positioning

·         Does NOT assist radiologists in performing minimally invasive procedures, such as angiography, embolization, or image-guided biopsies.

20. True or False: Performing laboratory tests on blood samples is one of the core tasks of a hospital lab technician.

·         True

·         False

21. Who is primarily responsible for diagnosing and prescribing treatment for medical conditions? (Physicians / Nurses / Pharmacists / Dietitians)

·         Pharmacists

·         Physicians

·         Dietitians

·         Nurses

Section IV: Exploring Collaborative Engagement: Assessing Frequency and Patterns of Collaborative Practices

For the following questions, please answer with either:

·         Never

·         Rarely

·         Occasionally

·         Frequently

22. How frequently do doctors engage in discussions with pharmacists regarding prescription validation based on lab results?

23. How frequently are medical meetings held where doctors and pharmacists discuss patient cases and medication plans?

24. In your healthcare setting, how often are pharmacists or clinical pharmacists present in different services alongside doctors?

25. Do pharmacists actively review and check medications for all patients within various services?

26. How often do doctors communicate with nurses to verify lab results, discuss symptoms, or address any patient complaints?

27. How often do doctors seek assistance from psychologists to help communicate a difficult or bad diagnosis to the patient?

Section V: Awareness and Willingness Towards Interprofessional Collaboration and Education (IPE)

28. Have you heard about Interprofessional Collaboration (IPC) and Interprofessional Education (IPE) before?

·         Yes, I am very familiar with both IPC and IPE.

·         Yes, I have heard of them but don't know much about them.

·         No, I have never heard of them.

29. Do you know the primary purpose of Interprofessional Collaboration (IPC) and Interprofessional Education (IPE)?

·         Yes, I am familiar with the concept.

·         No, I am not familiar with either concept.

·         I'm not sure.

30. In your opinion, what are the potential benefits of integrating Interprofessional Education (IPE) into the medical curriculum? (Select all that apply)

·         Improved teamwork and communication among healthcare providers

·         Enhanced patient-centered care

·         Better understanding of the roles of other healthcare professionals

·         Reduced medical errors

·         Increased job opportunities for medical graduates

·         I don't know anything about Interprofessional Education (IPE)

31. Would you be willing to participate in Interprofessional Education (IPE) activities as part of your medical curriculum?

·         Yes, I am very willing.

·         No, I am not willing.

·         I don't know.

32. Which types of Interprofessional Education (IPE) activities would you find most beneficial or interesting? (Select all that apply)

·         Interprofessional case-based discussions

·         Simulated patient care scenarios involving multiple healthcare professions

·         Collaborative research projects with students from other healthcare disciplines

·         Clinical rotations that include exposure to other healthcare professions

·         I am not sure.

33. On a scale of 1 to 5, with 1 being "Not Important" and 5 being "Very Important," how important do you believe Interprofessional Collaboration (IPC) and Interprofessional Education (IPE) are for the future of healthcare in terms of patient satisfaction, saving lives, reducing mortality, and enhancing the hospital's reputation?

34. Do you enjoy communicating with other healthcare professionals and value taking into consideration their advice? (Yes/No)

35. Do you collaborate with other healthcare professionals in your current role? This may include activities such as meetings, coaching, etc. (Yes/No/Not Applicable)

Section VI: IPEC Competency Self-Assessment Tool

Please answer the following with the most appropriate answer of the following:

·         Strongly Disagree

·         Disagree

·         Neither Agree or Disagree

·         Agree

·         Strongly Agree

36. I am able to choose communication tools and techniques that facilitate effective team interactions.

37. I am able to place the interests of patients at the center of interprofessional health care delivery.

38. I am able to place the interests of patients at the center of interprofessional health care delivery.

39. I am able to engage other health professionals in shared problem-solving appropriate to the specific care situation.

40. I am able to respect the privacy of patients while maintaining confidentiality in the delivery of team-based care.

41. I am able to inform care decisions by integrating the knowledge and experience of other professions appropriate to the clinical situation.

42. I am able to embrace the diversity that characterizes the health care team.

43. I am able to apply leadership practices that support effective collaborative practice.

44. I am able to respect the cultures and values of other health professions.

45. I am able to engage other health professionals to constructively manage disagreements about patient care.

46. I am able to develop a trusting relationship with other team members.

47. I am able to use strategies that improve the effectiveness of interprofessional teamwork and team-based care.

48. I am able to demonstrate high standards of ethical conduct in my contributions to team-based care.

49. I am able to use available evidence to inform effective teamwork and team-based practices.

50. I am able to act with honesty and integrity in relationships with other team members.

51. I am able to understand the responsibilities and expertise of other health professions.

52. I am able to maintain competence in my own profession appropriate to my level of training.

Section VII: Performance Assessment: Communication and Teamwork

Please answer the following with the most appropriate answer of the following:

·         Strongly Disagree

·         Disagree

·         Neither Agree or Disagree

·         Agree

·         Strongly Agree

53. I can work effectively in teams.

54. I can easily facilitate communication between team members.

55. I am not effective at delegating responsibility for tasks.

56. I am able to resolve conflicts between individuals effectively.

57. I do not feel I can take on a leadership role in a team and be effective.

Please answer the following with the most appropriate answer of the following:

·         Essential

·         Not Essential

·         Don’t Know

58. Collaboration

59. Working together to solve patients’ problems.

60. Reducing Errors

61. Improving quality of care

62. Anticipating the needs of other team members

63. Experiencing concerns about patient safety
